# Phase-contrast MRI analysis of cerebral blood and CSF flow dynamic interactions

**DOI:** 10.1186/s12987-024-00578-w

**Published:** 2024-10-28

**Authors:** Kimi Piedad Owashi, Pan Liu, Serge Metanbou, Cyrille Capel, Olivier Balédent

**Affiliations:** 1 Medical Image Processing Department, CHU Amiens-Picardie University Hospital, Amiens, France; 2 CHIMERE UR 7516, Jules Verne University of Picardy, Amiens, France; 3 Radiology Department, CHU Amiens-Picardie University Hospital, Amiens, France; 4 Neurosurgery Department, CHU Amiens-Picardie University Hospital, Amiens, France

**Keywords:** Cerebral blood, CSF, Volume change, Intracranial, Extracranial, Flow dynamics, Phase-contrast MRI

## Abstract

**Background:**

Following the Monro-Kellie doctrine, the Cerebral Blood Volume Changes (CB_VC) should be mirrored by the Cerebrospinal Fluid Volume Changes (CSF_VC) at the spinal canal. Cervical level is often chosen to estimate CB_VC during the cardiac cycle. However, due to the heterogeneity in the anatomy of extracranial internal jugular veins and their high compliance, we hypothesize that the intracranial level could be a better choice to investigate blood and cerebrospinal fluid (CSF) interactions. This study aims to determine which level, intracranial or extracranial, is more suitable for measuring arterial and venous flows to study cerebral blood and CSF dynamics interactions.

**Methods:**

The spinal CSF and cerebral blood flow measured at intracranial and extracranial levels were quantified using cine phase-contrast magnetic resonance imaging (PC-MRI) in 38 healthy young adults. Subsequently, CSF_VC and CB_VC were calculated, and by linear regression analysis (R^2^ and slope), the relationship between CB_VC at both levels and the spinal CSF_VC was compared. The differences between extracranial and intracranial measurements were assessed using either a paired Student’s t-test or Wilcoxon’s test, depending on the normality of the data distribution.

**Results:**

The CB_VC amplitude was significantly higher at the extracranial level (0.89 ± 0.28 ml/CC) compared to the intracranial level (0.73 ± 0.19 ml/CC; *p* < 0.001). CSF oscillations through the spinal canal do not completely balance blood volume changes. The R^2^ and the slope values obtained from the linear regression analysis between CSF and blood flows were significantly higher in magnitude for the intracranial CB_VC (R^2^: 0.82 ± 0.16; slope: − 0.74 ± 0.19) compared to the extracranial CB_VC (R^2^: 0.47 ± 0.37; slope: -0.36 ± 0.33; *p* < 0.001). Interestingly, extracranial CB_VC showed a greater variability compared to intracranial CB_VC.

**Conclusion:**

Our results confirmed that CSF does not completely and instantaneously balance cerebral blood expansion during the cardiac cycle. Nevertheless, the resting volume is very small compared to the total intracranial volume. To our knowledge, this study is the first to demonstrate these findings using cerebral blood flow measured intracranially below the Circle of Willis. Additionally, our findings show that cerebral arterial and venous flow dynamic measurements during the cardiac cycle obtained by PC-MRI at the intracranial plane strongly correlate with CSF oscillations measured in the spinal canal. Therefore, the intracranial vascular plane is more relevant for analyzing cerebral blood and CSF interactions during the cardiac cycle compared to measurements taken at the cervical vascular level.

## Introduction

According to the Monro-Kellie doctrine [1, 2], the total intracranial volume– comprising the brain parenchyma, cerebrospinal fluid (CSF), and blood– remains constant. Therefore, any volume changes in one compartment must be compensated by the others, with CSF playing a key role in maintaining this equilibrium to preserve mean intracranial pressure (ICP) within a physiological range, thus ensuring optimal brain perfusion.

The Monro-Kellie doctrine was initially formulated based on the hypothesis that the dura mater and all the intradural tissues are completely incompressible and that the tympanic membranes and the eyes globes are static. However, it has been shown [3] that small changes in total intracranial volume (less than 1 mL) do occur during each cardiac cycle. Alperin et al. [4] demonstrated that CSF oscillations through the spinal canal do not completely balance intracranial blood volume changes. Similarly, Miyati et al. [5] confirmed that intracranial volume change is not zero and is significantly smaller in idiopathic normal pressure hydrocephalus patients than in healthy volunteers. Therefore, while the Monro-Kellie doctrine is crucial for understanding intracranial dynamics over long periods and larger volumes (i.e., during infusion tests [6]), it may not be entirely applicable for assessing intracranial volume changes during the small scale of time and volume of one cardiac cycle.

Indeed, during each cardiac cycle, the interplay between the intracranial arterial inflow and its compensatory mechanisms: the intracranial compliance, the CSF flow oscillations, and the venous drainage, leads to ICP pulse changes [7, 8]. However, a remarkable observation during the cardiac cycle, consistent with the Monro-Kellie doctrine, is the mirroring effect where the dynamics of the Cerebral Blood Volume Changes (CB_VC) are reflected by the Cerebrospinal Fluid Volume Changes (CSF_VC) dynamics at the spinal canal. In fact, alterations in the dynamics of CSF and cerebral blood flow often lead to a wide range of cerebral disorders, including hydrocephalus [9, 10], syringomyelia [11, 12], Chiari malformations [13], Alzheimer’s disease [14], hyper or hypo intracranial pressure [15].

By the end of the 1990s, many studies [7, 16–18] using cine phase-contrast magnetic resonance imaging (PC-MRI), have demonstrated a strong correlation between spinal CSF and arterio-venous (AV) flow waveforms during the cardiac cycle, demonstrating the close hydrodynamic coupling of the brain blood and CSF flow within the skull over a cardiac cycle. Balédent et al. [8] suggested that this correlation is related to the expansion of the lumbar thecal sac and the compression of the epidural venous plexus, along with the large diameter of the spinal subarachnoid spaces, which allow extensive and instantaneous CSF venting mainly from the intracranial subarachnoid spaces through the foramen magnum in response to the arterial systole.

CSF pulsatile flow is generated by both the cardiac and respiratory cycles. In recent years, there has been growing interest in understanding cardiac- and respiratory-driven CSF pulsatility as a crucial component of cerebral homeostasis [19–22]. This pulsatility is influenced by cerebral arterial inflow and jugular outflow and may be affected by white matter abnormalities in various diseases, such as multiple sclerosis [23] and hypertension [24].

Most studies [25–29] consider the arterial and venous flows measured extracranially at the cervical level to assess cerebral blood flow dynamics. Cerebral arterial flow measurements show little variability between intracranial and extracranial levels. This consistency is observed in young [30] and older healthy adults [31, 32]. Conversely, some studies [31, 33, 34] have shown that the mean venous flow significantly differs when comparing intracranial and extracranial veins, presenting a higher variability in the venous flows measured at the internal jugular veins (IJVs). Moreover, these studies also reported a significantly higher pulsatility index in the flow measured extracranially at the IJVs than intracranially at the sinuses. Therefore, due to the significant heterogeneity in extracranial internal jugular veins anatomy [33, 35, 36], as well as variations in flow and higher compliance, we hypothesize that intracranial and extracranial vascular levels interact differently with spinal CSF.

This study aims to determine which level, intracranial or extracranial, is more suitable for measuring arterial and venous flows to study cerebral blood and CSF dynamics interactions. For this purpose, the spinal CSF and cerebral blood flow measured at intracranial and extracranial levels were quantified using cine PC-MRI in healthy young adults. Subsequently, CSF_VC and CB_VC were calculated, and by linear regression analysis, the relationship between CB_VC at both levels and the spinal CSF_VC was compared.

## Materials and methods

### Study populations

The study’s population consisted of 38 healthy young volunteers (HYV), 18 women and 20 men, with an average age of 25 ± 4 years, within an age range of 19–35 years. The participants did not present neurological, psychiatric, or severe general disease, alcoholism, or abnormalities detected by a clinical MRI exam.

Each participant underwent an MRI examination, which lasted about 30 min. The study was conducted in accordance with the principles outlined in the Declaration of Helsinki and approved by the local Ethics Review Committee (CPP Nord Ouest II, Amiens, France; reference: PI2019_843_0056). All participants were informed of the objectives and procedures of the study. Prior to their participation in the study protocol, all subjects provided written informed consent. The exclusion criteria included MRI contraindications and any cerebrovascular or respiratory disease history.

### PC-MRI acquisition

Participants were examined in the supine position, using a clinical 3T MRI system (Philips Achieva; maximum gradient = 80 mT/m; slew rate = 120 mT m^− 1^ ms^− 1^) equipped with a 32-channel head coil.

**Fig. 1:**
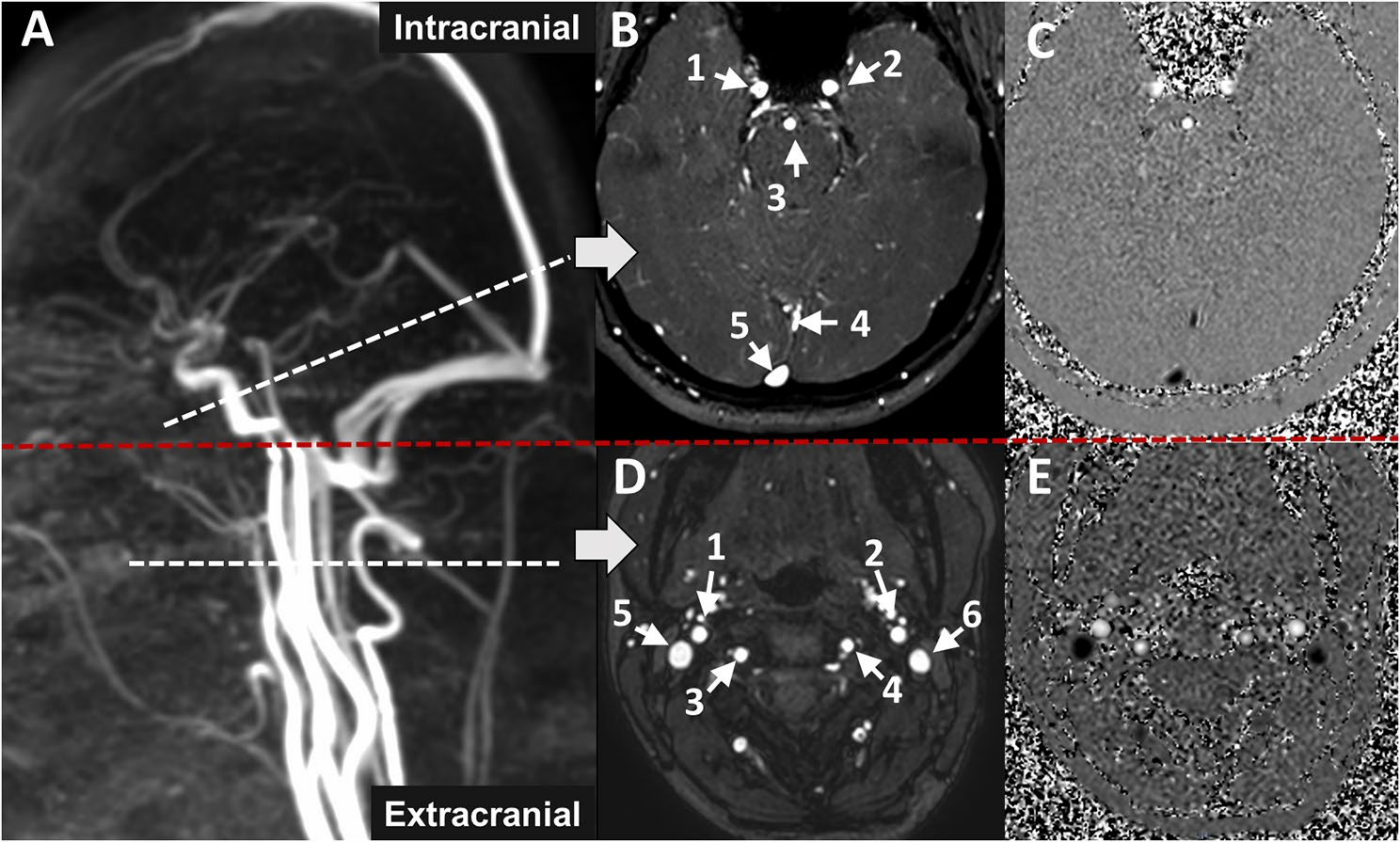
Extracranial and intracranial planes for blood flow measurements. Sagittal 3D phase-contrast angiography (**A**) was used as a reference to set the different acquisition planes perpendicular to the blood flow direction. (**B** and **C**), (**D** and **E**) represent the amplitude and phase images of the intracranial (extracranial) plane, respectively. In the phase images, white pixel intensity indicates flow in the positive direction, while black pixel intensity signifies flow in the negative direction. Flow directed towards the cranium is considered positive. Grey pixels represent areas of no flow. For the intracranial plane, 1: right internal carotid artery, 2: left internal carotid artery, 3: basilar artery, 4: straight sinus, 5: superior sagittal sinus. For the extracranial plane, 1: right internal carotid artery, 2: left internal carotid artery, 3: right vertebral artery, 4: left vertebral artery, 5: right internal jugular vein, 6: left internal jugular vein

Sagittal 3D phase-contrast cranio-cervical angiography (TE/TR = 3 ms/5 ms; FOV = 350 (FH) 248 (AP) 160 (RL) mm^3^; spatial resolution = 1.5 × 1.5 × 1.5 mm^3^; flip angle = 12°) was used as a reference to position the intracranial (below the Circle of Willis) and extracranial (high cervical spinal level) acquisition planes for cerebral arterial and venous blood flow measurements (Fig. 1).

The intracranial plane included three arteries: the left and right internal carotid arteries (ICAL_i and ICAR_i) and the basilar artery (BA), and two sinuses: the straight sinus (SS) and the superior sagittal sinus (SSS). The extracranial plane included four arteries: the left and right internal carotid arteries (ICAL_e and ICAR_e), as well as the left and right vertebral arteries (VAL and VAR), and two veins: the left and right internal jugular veins (IJVL and IJVR).

**Fig. 2:**
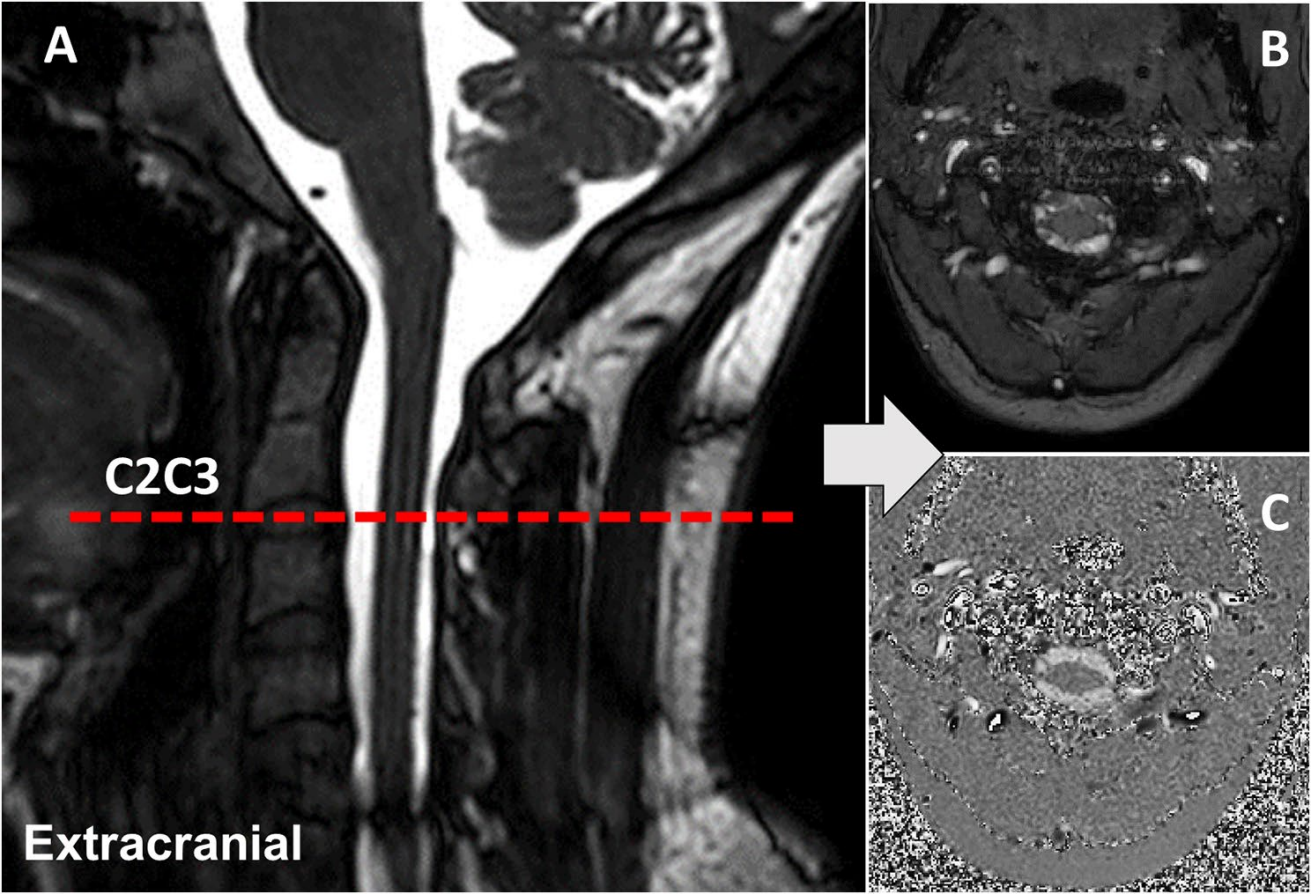
CSF flow measurement plane. Sagittal 3D balanced gradient echo sequence (**A**) was used to establish the C2C3 cervical spinal level for CSF flow measurement. (**B**) and (**C**) represent the amplitude and phase images at the C2C3 level, respectively. Note the presence of the denticulate ligament at this level

Additionally, the sagittal 3D balanced gradient echo sequence was used to establish the CSF flow measurement plane at the C2C3 cervical spinal level (Fig. 2). The settings for this sequence were as follows: TR = 5.5 ms, TE = 2.2 ms, FOV = 180 × 180 mm^2^, spatial resolution of acquisition = 0.6 × 0.6 × 1.2 mm^3^, and flip angle = 45°.

Thus, CSF flow at the C2C3 level, as well as arterial and venous blood flow at both extracranial and intracranial levels were quantified using a 2D PC-MRI sequence. The acquisition planes were selected perpendicular to the presumed direction of the blood and CSF flow. The flow direction towards the cranium is defined as positive and represented with white pixel intensity on the phase images. Reversely, the downward flow direction is considered negative and indicated by black pixel intensities. Grey pixels represent areas with no flow. Vascular and CSF velocities were acquired in separate acquisitions during the same session.

Table 1 presents the main settings used for the PC-MRI sequence for CSF and blood flow measurements. During the acquisition of cine PC-MRI images, a finger plethysmograph was used for retrospective cardiac gating. After 50–115 s of acquisition (depending on the subject’s heart rate), all the data were reconstructed into 32 phase-contrast images representing the flow rate variation within an average cardiac cycle (CC).

**Table 1:** Main parameters used for the CINE-PC sequence in CSF and blood flow measurements

Parameters	Blood (intracranial)	Blood (extracranial)	CSF
VENC (mm/s)	600	600	50
FOV (mm^2^)	142 × 142	120 × 120	120 × 120
Resolution (mm^2^)	1 × 1	1 × 1	0.8 × 0.8
Thickness (mm)	2	2	3
Flip angle (degree)	30	30	30
SENSE	1.5	1.5	1.5
TE (ms)	6–7	6–7	8–9
TR (ms)	10–11	10–11	13–14

VENC: velocity encoding; FOV: field-of-view; SENSE: sensitivity encoding; TE: echo time; TR: repetition time.

### Image processing

The PC-MRI images were post-processed using in-house software, “Flow” [8]. The software executes a semi-automatic segmentation algorithm for CSF and blood vessels delineation. The segmentation algorithm is based on temporal pixel intensity variation compared to the cardiac cycle frequency. Subsequently, a background region was defined to correct the eddy current effect. For this purpose, stationary tissue regions adjacent to the region of interest (ROI) were identified, and their average velocity was considered as the new zero velocity reference. Furthermore, the software includes a de-aliasing correction function [8] for instances where the neurofluid velocity exceeded the velocity encoding (VENC) parameter. For all segmented pixels in the ROI, 32 fluid velocity values are calculated over the CC. These values are then averaged, and multiplied by the ROI’s area to calculate the total dynamic flow rate across the ROI in the entire cardiac cycle.

**Fig. 3:**
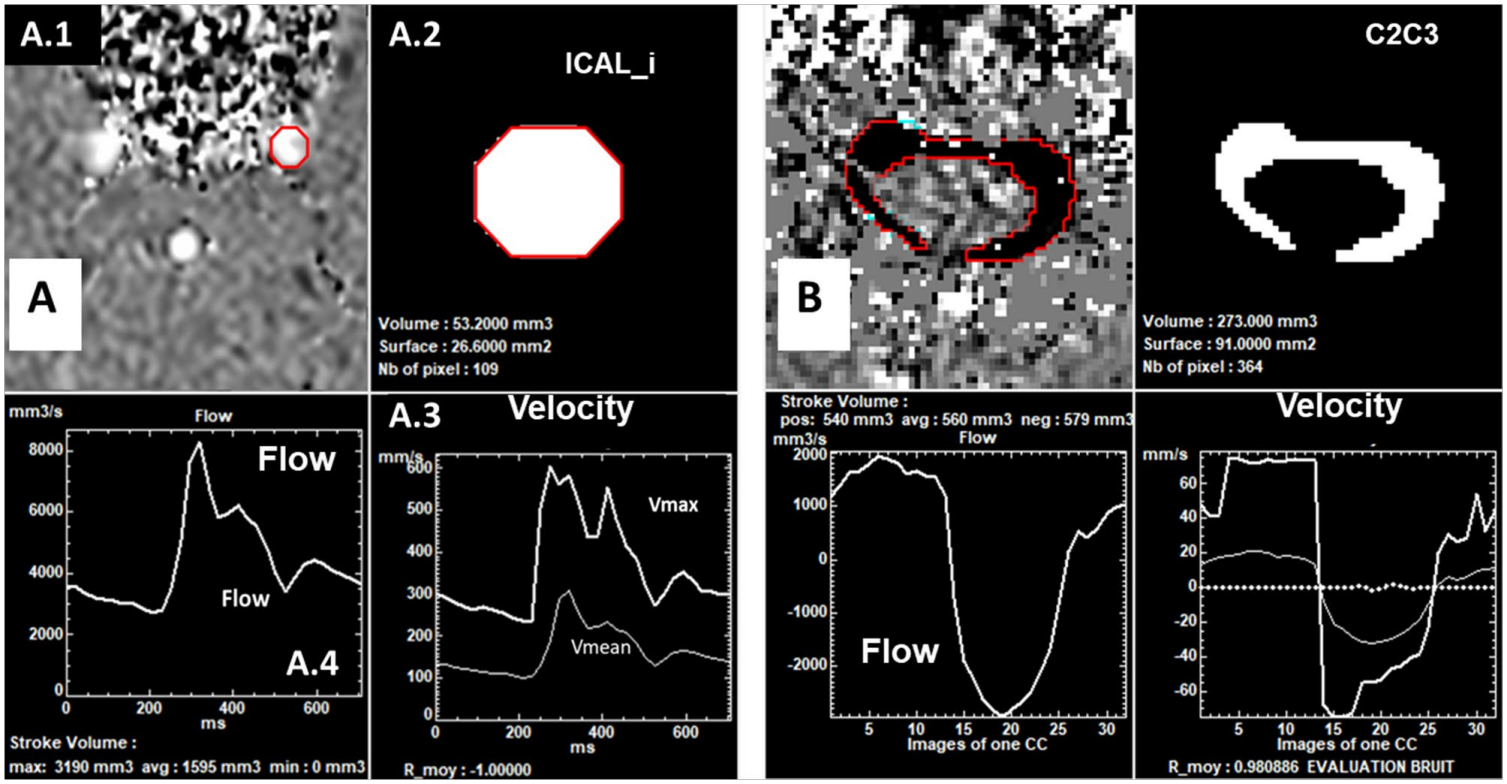
Examples of flow quantification using the “Flow” software. The software performs a semi-automatic segmentation and calculates the ROI area and the fluid velocity over the cardiac cycle. Subsequently, the software extracts the average flow waveform from the selected region. (**A**) Left internal carotid artery segmentation to calculate its area (A.2). Maximal and mean velocities curves dynamics over the cardiac cycle (A.3). Flow curve dynamic over the cardiac cycle (A.4). (**B**) Segmentation, velocities, and flow curve dynamics of CSF flow at the C2C3 level throughout the cardiac cycle

Thus, the software can extract the cerebral arterial and venous dynamic flow rate in the selected vessel, and the dynamic CSF flow rate at C2C3 (Fig. 3). The location and size of the ROI are assumed to be constant throughout the CC. Refer to previous studies [8, 37] for more details on PC-MRI image processing.

### Cerebral blood and CSF volume change dynamics

We calculated the total extracranial cerebral arterial blood flow curve (Qa_extra) by adding the flows curves from ICAL_e, ICAR_e, VAL, and VAR. Similarly, the total intracranial cerebral arterial blood flow (Qa_intra) was calculated by adding the flows curves from the ICAL_i, ICAR_i, and BA.

**Fig. 4:**
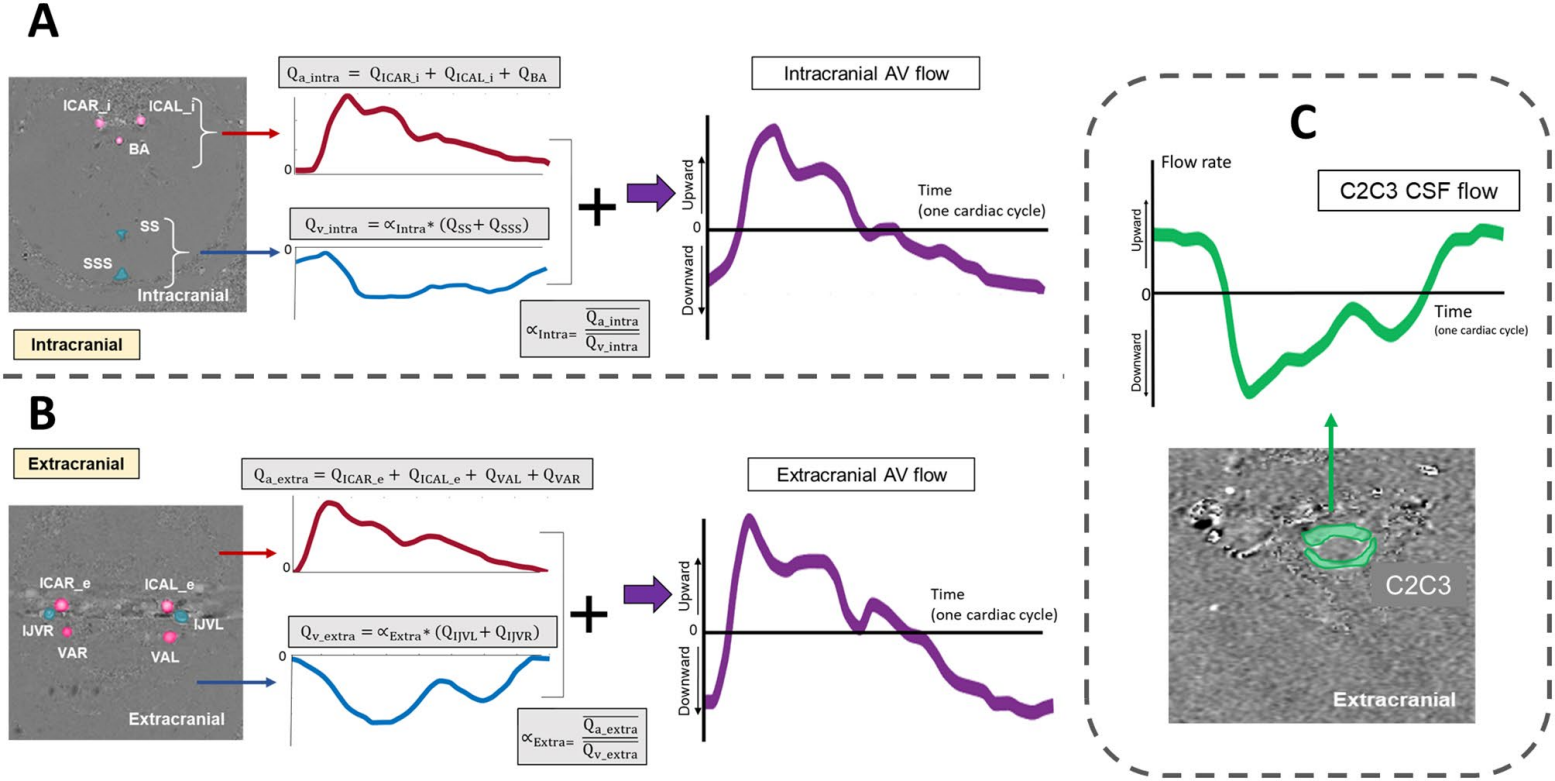
Cerebral blood and CSF flow curves. Phase-contrast imaging was used to quantify the total cerebral arterial (Qa) and venous (Qv) flow at the intracranial (**A**) and extracranial (**B**) levels. The measured venous flows, adjusted by the correction factors (αIntra, αExtra), resulted in the corrected total venous flows (Qv). The arterio-venous (AV) flow curves were then obtained by summing the total arterial and corrected venous flows at both intracranial and extracranial planes. (**C**) CSF flow curve obtained from phase-contrast imaging at the C2C3 level

The total extracranial venous blood flow curve (Qv_extra) and the total intracranial cerebral venous blood flow (Qv_intra) were calculated in two steps. First, we added IJVL and IJVR flows for the extracranial level, and for the intracranial level, we added the SS and SSS flows. To account for unconsidered peripheral cerebral venous drainage, we assumed that the mean Qv_extra equals the mean Qa_extra, and similarly, the mean Qv_intra equals the mean Qa_intra. Consequently, one correction factor for each level, αExtra and αIntra, were calculated by dividing the cycle-average total arterial flow value by the cycle-average venous flow value measured (Fig. 4A&B).

Then, Qv_extra and Qv_intra were calculated by multiplying the jugulars flow curve and the sinuses flow curve by αExtra and αIntra, respectively.

The arterial and venous pulsatility index (PI) was estimated at cervical and intracranial levels:

$$\:\text{P}\text{I}=\:\frac{\text{F}\text{l}\text{o}\text{w}\:\text{a}\text{m}\text{p}\text{l}\text{i}\text{t}\text{u}\text{d}\text{e}}{\text{M}\text{e}\text{a}\text{n}\:\text{f}\text{l}\text{o}\text{w}},$$

where the flow amplitude was calculated by subtracting the maximum and minimum flow values over the cardiac cycle.

Although cerebral arterial and venous flow curves, by construction, exhibit the same mean flow over the cardiac cycle, their dynamics throughout the cycle differ. Consequently, the cerebral blood volume changes (CB_VC) during the cardiac cycle were analyzed. To capture this, we calculated extracranial and intracranial arterio-venous (AV) flow curves by adding Qv_extra and Qv_intra to Qa_extra and Qa_intra, respectively (Fig. 4A&B). The CB_VC dynamics along 32 temporal points of the cardiac cycle were calculated at the two levels by integrating the AV flows over time.

**Fig. 5:**
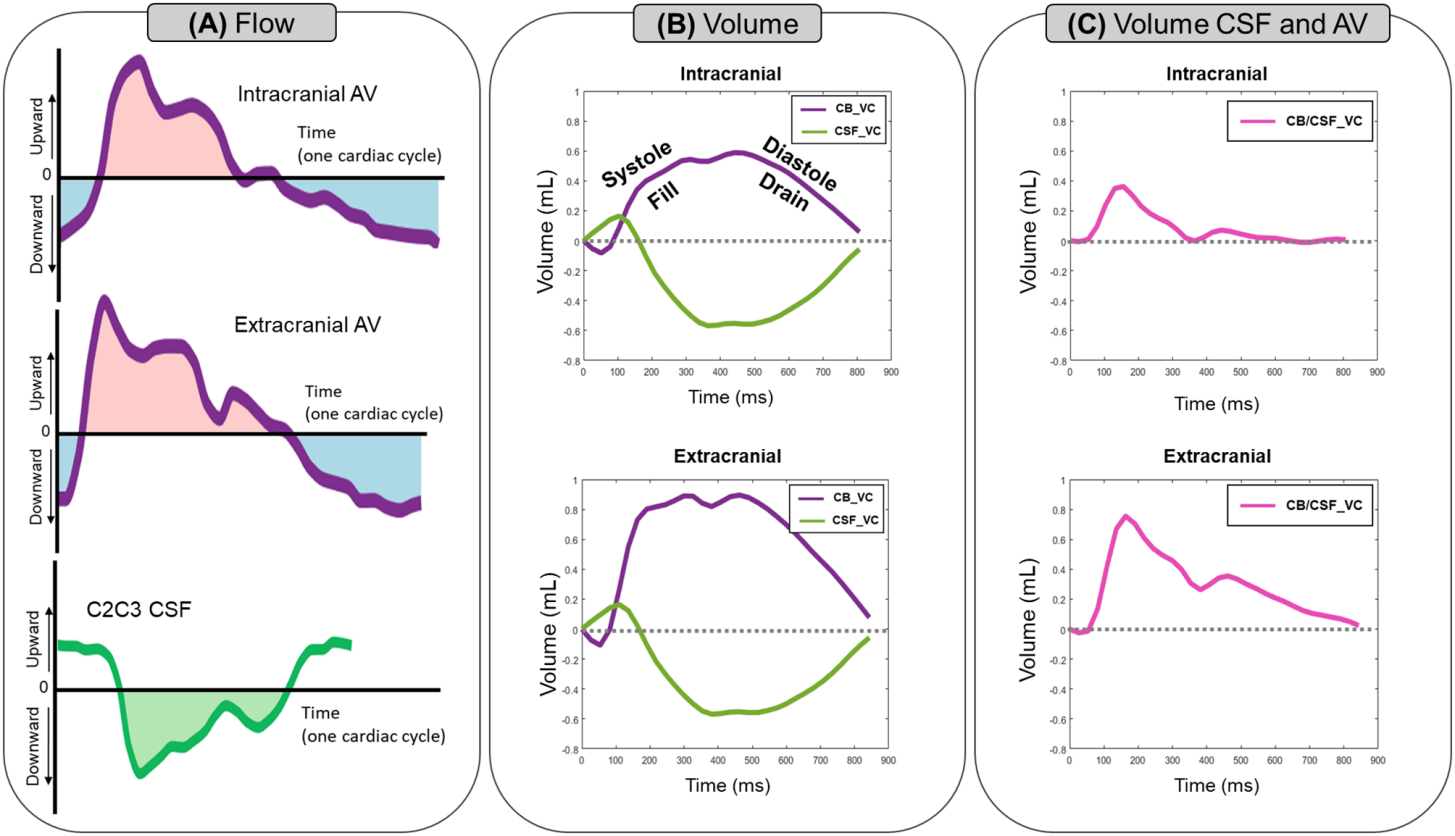
Cerebral Blood Volume Change (CB_VC) and CSF Volume Change (CSF_VC) curves. The CB_VC curves (*in purple, B*) throughout the cardiac cycle were calculated at the intracranial (*top*) and extracranial (*bottom*) levels by integrating the arterio-venous (AV) flows (*in purple, A*) over time. Moreover, the CSF_VC dynamic (*in green, B*) along the cardiac cycle, calculated by integrating the CSF flow measured at C2C3 (*in green, A*), was compared with the CB_VC dynamics calculated at both intracranial and extracranial planes. During systole, the volume of the arterio-venous compartments increases, indicating a filling phase. This expansion is mirrored by an increase in spinal CSF volume, a response to the changes in blood volume. Conversely, during diastole, the volume of arterio-venous compartments decreases, indicative of a draining phase, which corresponds with a decrease in spinal CSF volume. The CB_VC and CSF_VC curves were added to obtain the total intracranial volume change dynamics at both planes (*in pink, C*)

Due to the close relationship between AV flows and CSF flow at the spinal canal, we aimed to compare the dynamics of CB_VC with the CSF volume change over the cardiac cycle (CSF_VC). CSF_VC was calculated by integrating the CSF flow measured at the C2C3 level. The amplitudes of the CB_VC and CSF_VC curves, defined as the difference between their maximal and minimal values, were calculated to estimate the CSF and blood volumes oscillating through the intracranial compartments during the cardiac cycle. These amplitudes represent the stroke volume (SV). Furthermore, the blood and CSF volume change curves were added to obtain the total volume change dynamics (CB&CSF_VC) at each plane (See Fig. 5).

### Statistical analysis

The statistical analyses were performed with MATLAB software scripts (version 2019b, Mathworks, Natick, WA, USA). Descriptive statistics were expressed as the mean ± standard deviation (SD). Linear regressions were performed to evaluate the relationship (R^2^ and slope) between the 32 points of the CSF_VC curve and the 32 points of the two CB_VC curves (intracranial and extracranial planes). The Shapiro-Wilk test was used to determine whether the data were normally distributed. The differences between extracranial and intracranial measurements were assessed using either a paired Student’s t-test or Wilcoxon’s test, depending on the normality of the data distribution. The percentage of the coefficient of variation (CV%) was calculated by dividing the standard deviation by the mean and multiplying the result by 100. Correlations were assessed with Pearson’s test (for normal distributions) or Spearman’s test. The threshold for significance was set to *p* < 0.05.

## Results

**Fig. 6:**
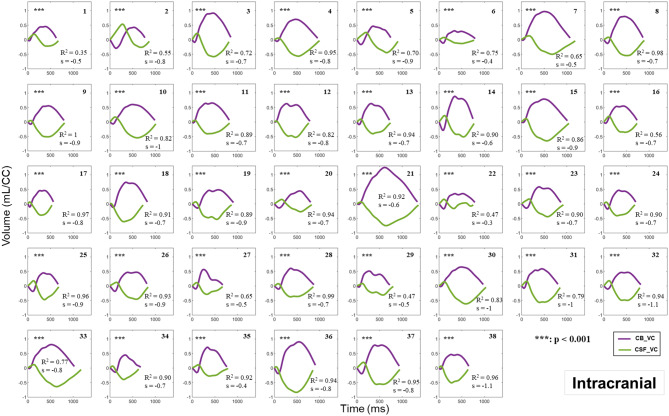
CB_VC (intracranial) and CSF_VC in the 38 participants. The CB_VC dynamics (*in purple*) at the intracranial level are compared with the CSF_VC dynamics (*in green*) at the C2C3 level along the cardiac cycle (CC). The coefficient of determination (R^2^) and the slope value (s), derived from linear regression analysis conducted to evaluate the relationship between the 32 points of the CSF_VC curve and the 32 points of the intracranial CB_VC curve, are displayed. ***: *p* < 0.001

**Fig. 7:**
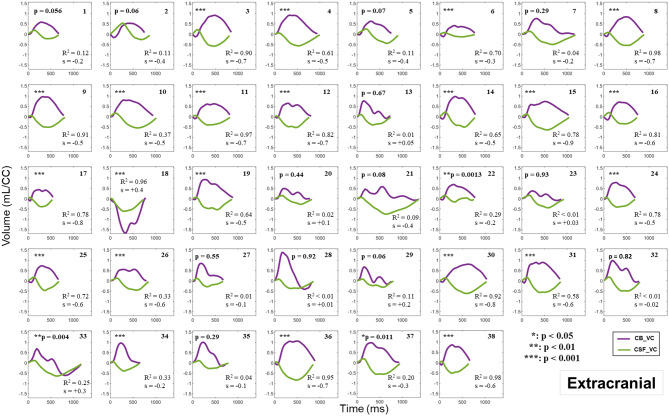
CB_VC (extracranial) and CSF_VC in the 38 participants. The CB_VC dynamics (*in purple*) at the extracranial level are compared with the CSF_VC dynamics (*in green*) at the C2C3 level along the cardiac cycle (CC). The coefficient of determination (R^2^) and the slope value (s), derived from linear regression analysis conducted to evaluate the relationship between the 32 points of the CSF_VC curve and the 32 points of the extracranial CB_VC curve, are displayed. *: *p* < 0.05; **: *p* < 0.01; ***: *p* < 0.001

Figures 6 and 7 depict the relationship between the CB_VC and CSF_VC curves of the 38 participants at the intracranial and extracranial levels, respectively, during the cardiac cycle. The curves in purple illustrate the CB_VC dynamics, with the intracranial AV dynamics presented in Fig. 6 and the extracranial AV dynamics in Fig. 7. The curves in green represent the CSF_VC dynamics, measured at the C2C3 level. Notably, these green curves are identical in Figs. 6 and 7 for each participant. Moreover, these figures present the results from the linear regression analysis performed between each participant’s CB_VC and CSF_VC data; hence, R^2^ denotes the coefficient of determination, ‘s’ represents the slope value of the linear equation and p the p-value.

**Fig. 8:**
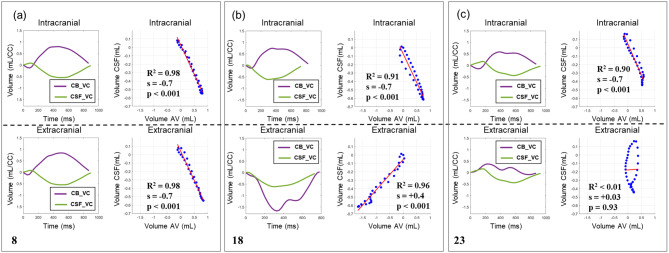
Examples from linear regression analysis. Results from three different subjects. CB_VC (*in purple*) and CSF_VC (*in green*) curves along the cardiac cycle (CC) with corresponding intracranial and extracranial linear regressions. For each case, the results of R^2^, slope and p-values are displayed. (**a**) A strong and negative linear relationship in both planes; (**b**) a strong, yet positive, linear relationship at the extracranial plane; (**c**) a non-significant extracranial relationship

In Fig. 8, examples from three different subjects are shown. For the first example (Fig. 8-a, participant #8), the CB_VC and the CSF_VC presented a strong negative linear relationship at both intracranial (R^2^: 0.98; slope: -0.7; *p* < 0.001) and extracranial (R^2^: 0.98; slope: -0.7; *p* < 0.001) levels. In the second case (Fig. 8-b, participant #18), both planes exhibited strong relationships, as evidenced by high R^2^ values (Intracranial R^2^: 0.91; Extracranial R^2^: 0.98); however, the slope value at the extracranial plane was positive (Extracranial slope: +0.4; *p* < 0.001). This positive slope value indicates a positive relationship between CB_VC and CSF_VC dynamics, suggesting a loss of the typical mirroring effect between cerebral blood and spinal CSF dynamics. The last example (Fig. 8-c, participant #23) demonstrated a strong and negative intracranial linear relationship (R^2^: 0.90; slope: -0.7; *p* < 0.001), in contrast to a non-significant extracranial linear relationship (R^2^ < 0.01; slope: +0.03; *p* = 0.93).

The results of CSF and cerebral blood flow dynamics at the intracranial and extracranial planes are summarized in Table 2. Significant differences were observed between the extracranial and intracranial arterial mean flow (*p* < 0.001) and venous mean flow (*p* = 0.014). Similarly, for the arterial and venous pulsatility indexes (*p* < 0.001). However, the correction factors αExtra and αIntra did not differ significantly (*p* = 0.57). It is important to highlight that the mean αExtra factor was calculated excluding participant #28, who was considered an outlier, as only 4% of mean venous flow accounted for mean arterial inflow. Specifically for this participant, the venous drainage was primarily redirected into the epidural and posterior veins, a measurement that was not included in this study.

Concerning the volume change dynamics, interestingly, all the measurements taken extracranially showed a greater variability compared to those taken intracranially. The CB_VC amplitude was significantly higher at the extracranial level (0.89 ± 0.28 ml/CC) compared to the intracranial level (0.73 ± 0.19 ml/CC; *p* < 0.001). The R^2^ and the slope values obtained from the linear regression analysis were significantly higher in magnitude for the intracranial CB_VC (R^2^: 0.82 ± 0.16; slope: − 0.74 ± 0.19) compared to the extracranial CB_VC (R^2^: 0.47 ± 0.37; slope: -0.36 ± 0.33; *p* < 0.001).

**Table 2:** Comparison of intracranial and extracranial levels

Parameters (*n* = 38)	Intracranial		Extracranial		
	Mean ± SD	CV (%)	Mean ± SD	CV (%)	p-value
**Flow**					
Cerebral arterial mean flow (ml/min)	659 ± 124	19	725 ± 128	18	< 0.001^***^
Measured cerebral venous mean flow (ml/min)	450 ± 95	21	511 ± 170	33	0.014^*^
Correction factor α	1.63 ± 1.15	71	1.62 ± 1.03	64	0.57
PI arterial	0.81 ± 0.15	18	0.95 ± 0.23	24	< 0.001^***^
PI venous	0.25 ± 0.07	28	0.58 ± 0.32	55	< 0.001^***^
**Volume change**					
Amplitude CB_VC (ml/CC)	0.73 ± 0.19	26	0.89 ± 0.28	31	< 0.001^***^
Amplitude CSF_VC (ml/CC)	-		0.59 ± 0.16	27	-
Amplitude CB&CSF_VC (ml/CC)	0.34 ± 0.13	38	0.77 ± 0.47	61	< 0.001^***^
**Linear regression**					
R^2^	0.82 ± 0.16	19	0.47 ± 0.37	79	< 0.001^***^
Slope	− 0.74 ± 0.19	26	-0.36 ± 0.33	92	< 0.001^***^

Values are presented as means ± standard deviation (SD). The amplitudes of the volume change curves represent the stroke volume. All data were tested for their distribution’s normality, followed by a comparison of intracranial and extracranial levels using a paired Student’s t-test or Wilcoxon’s test (*: *p* < 0.05; **: *p* < 0.01; ***: *p* < 0.001). PI: pulsatility index of the total arterial cerebral blood flow and total corrected cerebral venous flow; CC: cardiac cycle; R^2^: coefficient of determination; CV: coefficient of variation

**Fig. 9:**
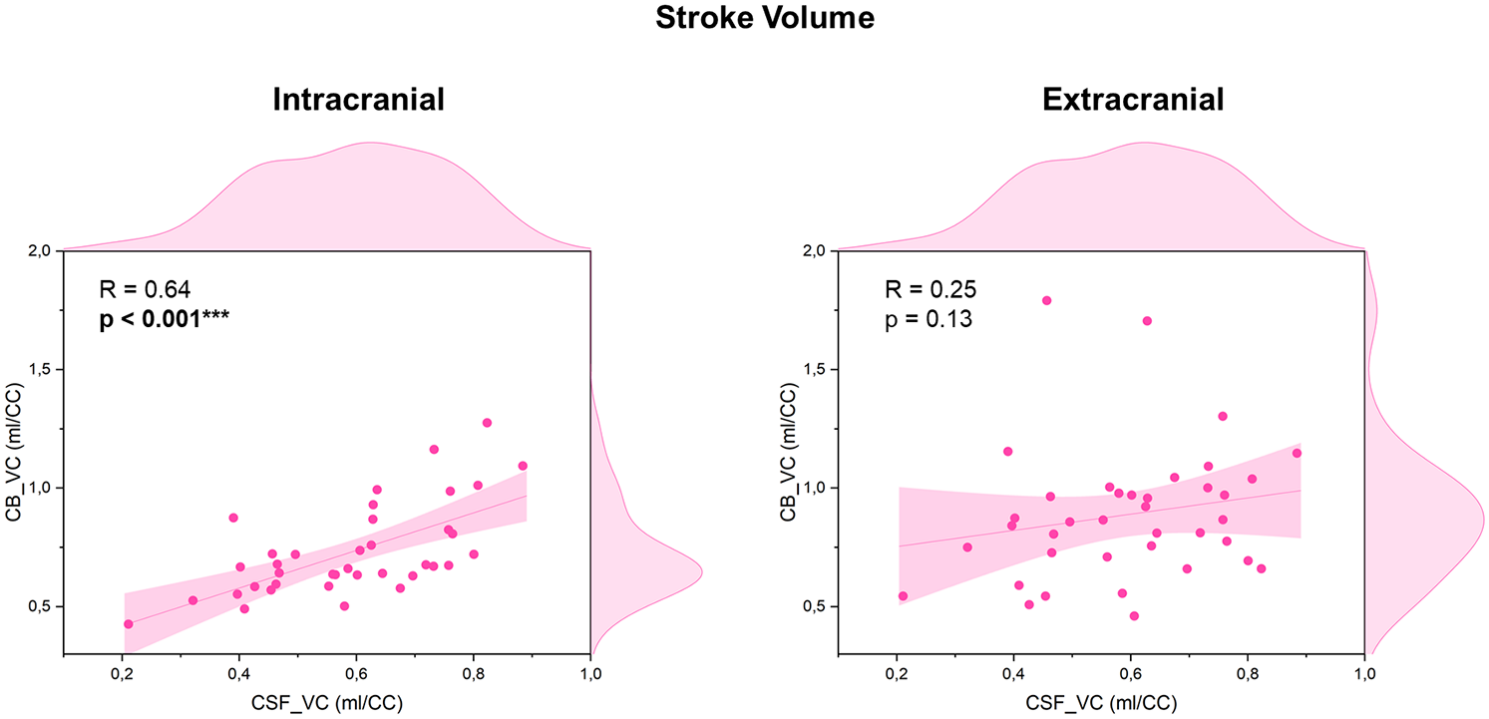
Correlation between cerebral blood and CSF stroke volume. On the *left*, the correlation between the stroke volumes of the spinal CSF and the intracranial cerebral blood. On the *right*, the correlation between the stroke volumes of the spinal CSF and the extracranial cerebral blood. R and p correspond to Spearman’s coefficient and the p-value of the correlation (***: p-value < 0.001)

Figure 9 illustrates the correlations between spinal CSF and cerebral blood stroke volumes at both intracranial and extracranial levels. A strong correlation is evident between the amplitudes of the intracranial CB_VC and the CSF_VC (*R* = 0.64, *p* < 0.001). In contrast, no significant correlation was found between the extracranial CB and spinal CSF stroke volumes (*R* = 0.25, *p* = 0.13).

## Discussion

This study aimed to compare the relationship between the CB_VC dynamics at intracranial and extracranial levels and the dynamics of spinal CSF_VC. The volume change dynamics were derived from arterial, venous and CSF flow measurements obtained from cine PC-MRI sequences of healthy young adults.

As presented in Table 2, the mean arterial flow was significantly higher at the extracranial level than at the intracranial level. Indeed, the paired vertebral arteries at the extracranial plane, besides basilar artery supply, also provide blood to the cerebellum arteries. However, the blood flow in cerebellum arteries was not included in the measurement approach described in this study. Balédent et al. [3] reported that about 13.5% of extracranial arterial blood volume reached the cerebellum in healthy young adults. Furthermore, in a prior study [32], we estimated that the volume of blood supplying the cerebellum’s arterial vascularisation is about 8% for healthy young volunteers and 0.5% for healthy elderly volunteers. The percentage of blood volume reaching the cerebellum is not negligible, especially in younger adults; still, we hypothesise that cerebellar vascularisation does not influence the haemodynamic between the extracranial and intracranial compartments and is therefore not taken into account.

Unlike cerebral arteries, which have a consistent anatomical pattern in all individuals, cerebral veins show considerable variability in their anatomy, making them more challenging to study. Table 2 reveals a higher venous flow and PI at the extracranial plane than at the intracranial plane. More interestingly, extracranially, the venous variability in flow and pulsatility was remarkably higher, contrasting with the variability observed in arterial flow and PI between both planes.

At the intracranial plane, the dural sinuses demonstrate variation in both anatomy [36, 38] and flow patterns [35]. Indeed, the straight and superior sagittal sinuses were computed separately, rather than measuring a single flow through the torcular Herophili, due to the significant variability in its configuration across individuals [39–41] and the high turbulence present in this region, which can affect measurement accuracy.

Extracranial internal jugular veins exhibit even more considerable flow and anatomical structure variability. Previous studies [31, 33, 34] have consistently shown that the pulsatility index in the internal jugular veins (IJVs) is significantly higher and more variable than at the SSS and SS, consistent with the findings of our present study. The IJVs are considered the primary venous drainage pathway, at least in the supine position [42]. Several studies have shown that cerebral venous outflow distribution depends on body posture [43] and central venous pressure [42]. However, Stoquart-ElSankari et al. [33] observed that, although IJVs flow was predominantly significant in a study involving 18 healthy young participants in supine position, in four of these volunteers, the cephalic venous drainage was dominantly or exclusively diverted into the epidural veins, despite the absence of any pathological findings or abnormalities in the MRI results. In this study, for example, participant #28 exhibited venous drainage that predominantly occurred through two epidural veins and three posterior veins, in contrast to the flow through the IJVs, which accounted for only 4% of the arterial inflow. Additionally, Stoquart-ElSankari et al. observed that eight healthy volunteers in their study population presented strictly unilateral IJV drainage of cerebral venous blood without any contralateral IJV flow. In the present study, seven HYVs exhibited unilateral IJV flow.

This study assessed the entire subarachnoid space surrounding the spinal cord instead of being segmented into anterior and posterior compartments. The presence of the denticulate ligament, which may vary depending on the specific slice level selected for image acquisition, does not significantly influence the overall CSF flow measurement.

The main interest of this study was to compare the relationship between the cerebral blood dynamics, measured extracranially and intracranially, and the spinal CSF dynamics. The present study revealed several findings.

First, by integrating cerebral blood and CSF flows, we calculated the total volume change (CB&CSF_VC) at each plane. Contrary to the Monro-Kellie doctrine’s assertion, our findings indicate that the total intracranial volume within one cardiac cycle is not zero. Instead, the volume change varies depending on the measurement location, averaging 0.34 ml/CC when measured intracranially and 0.77 ml/CC when measured extracranially. While this finding is consistent with prior studies [5, 28, 29] that considered extracranial cerebral blood measurements, to our knowledge, this study is the first to quantify the intracranial volume change using arterial and venous flows measured intracranially below the Circle of Willis.

Second, as shown in Table 2, the amplitude of CB_VC significantly differs between the two planes, suggesting that the cerebral blood dynamics is variable and dependent on the measurement acquisition plane. As expected, the CB_VC amplitude is more variable at the extracranial plane than at the intracranial plane, given that, as previously mentioned, the venous flow within the IJVs also exhibits high variability.

Third, the linear regression analysis results reveal a stronger linear relationship between spinal CSF_VC and intracranial CB_VC. Additionally, linear regression parameters presented higher variability at the extracranial level. As illustrated in Figs. 6 and 7, the CSF_VC dynamics generally attempt to mirror those of the CB_VC. However, there are notable exceptions, specifically in participants # 18 and 33, where significant (*p* < 0.05) positive relationships between CSF_VC and CB_VC were observed exclusively at the extracranial level, indicating a deviation from the typical mirroring dynamic. It is important to note, however, that this phenomenon is rare; out of the 38 cases, only two exhibited this behavior. While the mirroring effect between the CSF and the cerebral blood dynamics is consistently evident at the intracranial plane, the extracranial plane presents a range of variations, as demonstrated in Fig. 8. The correlation between the cerebral blood and CSF stroke volumes (Fig. 9) reiterates the significant correlation of spinal CSF_VC with the intracranial CB_VC, in contrast to the extracranial CB_VC.

These findings suggest that the CSF response to vascular volume variation within the craniospinal system is more consistent with the arterial and venous measurements acquired at the intracranial plane. Notably, the differences observed between both levels are mainly attributed to the venous compartment, as extracranially, the veins system shows greater inter-subject variability and complexity in vessel morphology and flow pulsatility. Rapidly, because of the compliance of the internal jugular veins, blood volume can easily expand outside the cranium, modifying the dynamics of the cerebral blood volume curve.

The coefficient of determination (R^2^) and the slope are indicative of the linearity of volume exchange between the cerebral blood and CSF compartments, as well as the temporal relationship in this exchange. The rapid arterial inflow during systole leads to a rise in ICP pulses, which rapidly induces a CSF flush into the spinal subarachnoid spaces [3]. Thus, despite the evident variability in venous compartments, a time lag between arterial or venous flow dynamics in the two planes could also influence the linearity of volume change dynamics. Although not statistically significant, our previous study [32] reported a longer upstroke time for intracranial arterial flow compared to extracranial arterial flow, suggesting the potential time lag between arterial flows in the two planes, which could also affect the CSF response.

Increased ICP has been reported in several neurological diseases, including hydrocephalus, idiopathic intracranial hypertension, and traumatic brain injury [44]. The study of cerebral blood and CSF interactions is essential to understand the mechanisms underlying the ICP pulse variations. Consequently, the ICP should be more closely related to intracranial AV than extracranial AV measurements.

To our knowledge, there are currently no studies comparing the relationship between intracranial and extracranial cerebral blood dynamics with spinal CSF dynamics. Our findings confirmed that AV dynamics differ depending on the measurement acquisition plane. This suggests that the intracranial plane is more suitable for measuring arterial and venous flows when studying the interactions between cerebral blood and CSF dynamics.

### Limitations

In the present study, we did not consider the effects of blood pressure, gender or height on the vascular-CSF flow dynamics. Nevertheless, we believe that these parameters would not significantly impact our results.

The presence of air produces a high susceptibility to artefacts, particularly in regions close to the paranasal sinuses there are significant field inhomogeneities that can distort the image [45]. Acquisitions at the intracranial level were obtained close to the nasal cavity, which could produce high susceptibility to image artefacts indeed. However, in this study, the artifacts caused by air were relatively minor, and their impact on the accuracy of the blood flow measurements was minimal.

While peripheral gating was chosen for consistency and practicality, we acknowledge that it may introduce some limitations in accuracy compared to ECG gating [46]. However, we carefully optimized its use to minimize potential errors.

It is important to highlight that cerebral blood and CSF flows are not acquired simultaneously during the imaging protocol. Consequently, participants’ heart rates could vary between acquisitions, which could affect the linear relationship outcomes between CB_VC and CSF_VC. Nonetheless, throughout our protocol, care was taken to ensure that heart rate variations did not differ significantly between acquisitions. On the other hand, Figs. 6 and 7 illustrate the wide range of variability in cardiac cycle length among participants. As observed in a previous study [32], this variability is mainly due to changes in diastole duration. While systole duration remains nearly preserved, the diastole duration is adjusted according to the heart rate. In future studies, it might be interesting to analyze whether the cardiac cycle duration impacts CSF and blood flow dynamics.

## Conclusion

The results of the present study offer valuable reference values of cerebral vascular-CSF flow interaction in young healthy adults.

First, we have shown that CSF does not completely and instantaneously balance intracranial blood expansion during the cardiac cycle, as expected by the Monro-Kellie doctrine. Nevertheless, the resting volume is very small compared to the total intracranial volume. To our knowledge, this study is the first to demonstrate these findings using cerebral blood flow measured intracranially below the Circle of Willis.

This study aimed to determine which level, intracranial or extracranial, is more suitable for measuring cerebral arterial and venous flows to study cerebral blood and CSF dynamics interactions. Our results showed that cerebral arterial and venous flow dynamic measurements during cardiac cycle obtained by PC-MRI at the intracranial plane strongly correlate with CSF oscillations measured in the spinal canal, with R^2^ values ranging from 0.35 to 1. Moreover, correlation was significantly weaker when cerebral blood flows was measured at the cervical level. Intracranial vascular plane is more relevant for analyzing the interaction between cerebral blood and CSF compared to the extracranial vascular level, where significant individual venous heterogeneity exists.

## Data Availability

No datasets were generated or analysed during the current study.

## Abbreviations

AV: Arterio-venous
BA: Basilar artery
CB_VC: Cerebral blood volume change
CC: Cardiac cycle
CSF: Cerebrospinal fluid
CSF_VC: Cerebrospinal fluid volume change
FOV: Field of view
HYV: Healthy young volunteer
ICP: Intracranial pressure
ICAL: Left internal carotid artery
ICAR: Right internal carotid artery
IJV: Internal jugular vein
IJVL: Left internal jugular vein
IJVR: Right internal jugular vein
PC-MRI: Phase-contrast magnetic resonance imaging
PI: Pulsatility index
SENSE: Sensitivity encoding
ROI: Region of interest
SS: Straight sinus
SSS: Superior sagittal sinus
SV: Stroke volume
TE: Echo time
TR: Repetition time
VAL: Left vertebral artery
VAR: Right vertebral artery
VENC: Velocity encoding
